# Communication With Older Adults in Times of a Pandemic: Practical Suggestions for the Health Care Professionals

**DOI:** 10.3389/phrs.2021.1604046

**Published:** 2021-05-27

**Authors:** Alexis Pinsonnault-Skvarenina, Adriana Bender Moreira de Lacerda, Mathieu Hotton, Jean-Pierre Gagné

**Affiliations:** ^1^ School of Speech-Language Pathology and Audiology, Faculty of Medicine, University of Montreal, Montreal, QC, Canada; ^2^ Center for Interdisciplinary Research in Rehabilitation of Greater Montreal (CRIR), Montreal, QC, Canada; ^3^ Research Center of the Institut universitaire de gériatrie de Montréal (CRIUGM), Montréal, QC, Canada; ^4^ School of Rehabilitation, Faculty of Medecine, Université Laval, Québec, QC, Canada; ^5^ Center for Interdisciplinary Research in Rehabilitation and Social Integration (CIRRIS), Québec, QC, Canada; ^6^ Titulaire de la Chaire de la Fondation Caroline-Durand en audition et vieillissement de l'Université de Montréal, Montréal, Québec, QC, Canada

**Keywords:** COVID-19, communication, hearing loss, face mask, health care

## Abstract

In order to limit the spread of the coronavirus, several protective measures have been put in place in the community, in private and public residences and in health care centers. Some measures have a negative impact on communication. They include physical distancing, the use of face masks and shields as well as the increased use of telephone and videoconferencing for distance communication. The effects of COVID-19 are particularly harsh on older adults. Consequently, older adults, especially those with hearing loss, are particularly at risk of experiencing communication breakdowns and increased social isolation. Health care professionals should learn about and be encouraged to use communication strategies to maintain good interactions with their patients. This article proposes practical suggestions to health professionals who interact with older adults, especially those who have difficulty understanding speech. The goal of this article is to inform on the prevalence of hearing loss, the hearing difficulties experienced by older adults, the manifestations of hearing problems, the effects of pandemic protection measures on communication and the strategies that can be used to optimize professional-patient communication during a pandemic.

## Background

Coronaviruses are known to cause respiratory damage. A new strain of coronavirus, SARS-CoV-2 (Severe Acute Respiratory Syndrome Coronavirus 2), was identified in December 2019. The term COVID-19 refers to the infection caused by the virus, Coronavirus Disease 2019. Current scientific data shows that the virus is transmitted mainly by droplets of respiratory secretions (e.g., coughing, sneezing, and speaking). In order to limit the spread of the virus, several protective measures have been put in place in the community, private and public residences, as well as in health care centers. In addition to hand washing, social isolation and telecommuting, physical distancing and the use of a face mask, face shield and transparent partitions are recommended by public health agencies around the world [1].

Older individuals with hearing loss, whether they use hearing aids or not, learn to employ a variety of strategies to optimize speech understanding and communication [2–4]. For example, one strategy involves integrating visual speech cues (speechreading) and the auditory speech cues produced by the talker. In degraded communication settings, using visual speech cues may improve speech intelligibility by as much as 40% [5] or have the same effect as improving the signal-to-noise ratio (SNR) by 8–11 dB [6,7]. Some measures proposed to counter the spread of COVID-19 have a negative impact on communication regardless of whether or not the individuals involved have hearing loss. For example, some of the proposed measures may result in hiding the lips and part of the face of the talker or they may interfere with the propagation of the acoustic signal generated by the talker. The negative impact on speech communication may be considerable and thus may have a deleterious effect on activities of daily living and on social and professional integration of people with hearing loss, especially older adults. Also, it may have a negative impact on the health care services they receive and on their adherence to the treatment regimen that is recommended for them. The COVID-19 pandemic brought about changes in the habits and social activities of almost everyone, including older individuals. For example, in-person social contacts have decreased and the use of distance communication involving technology (e.g., email, telephone, videoconferencing app) has increased appreciably.

In times of pandemic, special consideration should be given to communication among the population of older individuals with hearing loss. Communication with COVID-19 protection measures is problematic. Health care professionals should be aware of this and use strategies designed to optimize professional-patient communication. Effective communication is a shared responsibility; it is not problematic only for persons with hearing loss.

This article is intended for all health care professionals who interact and provide services to older adults that experience difficulties understanding speech. The goal of this article is to provide practical strategies that can be used to optimize communication during the pandemic. The prevalence of hearing loss, the communication difficulties, the other manifestations of hearing problems and the effects of pandemic protection measures on communication are addressed. Although the information provided in the article is relevant for all adults with hearing loss, we will concentrate specifically on older adults.

## Hearing and Communication Difficulties of Older Adults With Hearing Loss

According to the World Health Organization [8], there are 466 million persons in the world with disabling hearing loss (6.1% of the world's population). Due to the increased longevity of individuals in most countries, the number of people with disabling hearing problems will continue to increase in the future. Projections show that the number could rise to 630 million by 2030 and may be over 900 million in 2050 [8]. The proportion of older adults with a hearing disability increases with age; while about 33% of adults over 65 years of age present with hearing loss [9], this proportion is more than twice as much among adults over 80 years of age—roughly 80% [10]. For older adults in nursing homes, it is estimated that the proportion of residents with hearing loss is over 80% [11].

**FIGURE 1:**
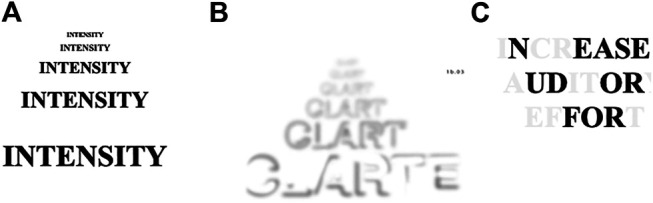
Visual analogy illustrating the consequences of age-related hearing loss. Legend: Panel **(A)**: Degree of hearing loss and loudness recruitment, Panel **(B)**: Poorer frequency resolution, and Panel **(C)**: Increased listening effort. This figure was adapted from Caron [16].

Presbycusis, or age-related hearing loss (ARHL), is defined as an age-related elevation of hearing thresholds [12]. This hearing loss is associated with many changes in the auditory system, including at the level of the sensory cells of the inner ear, the stria vascularis, the auditory nerve and the spiral ganglion [13]. It is believed that ARHL does not have a single cause but originates from an interaction of several factors. These factors may be intrinsic (e.g., gender, genetic diseases and disorders, systemic diseases, high blood pressure and metabolic diseases) or extrinsic (e.g., noise exposure, ototoxic medication, smoking and diet) to the aging individual [13–15]. Clinically, ARHL manifests itself by a progressive decrease in hearing detection thresholds that initially affects the higher audiometric frequencies, and then progressively spreads to the lower frequencies. A visual analogy of ARHL is illustrated in Figure 1. One consequence of ARHL is a decrease in the intensity of soft auditory signals coded by the auditory nerve (see Figure 1, panel A). Another consequence is a reduction in the clarity of the auditory message (see Figure 1, panel B). As a result of a loss in hearing sensitivity as well as spectral, temporal and other types of auditory distortions, speech understanding becomes less “automatic” and additional cognitive resources are required to decode and interpret the spoken message (see Figure 1, panel C). There is undeniable evidence that the use of hearing aids significantly improves speech understanding and reduces the psychosocial consequences of ARHL [17]. Thus, it is highly recommended that individuals who have hearing loss use hearing aids. Notwithstanding this recommendation, the focus of the present article is to describe other strategies to optimize speech understanding when protective measures are used in a pandemic.

Many older adults report negative psychosocial and emotional consequences attributable to their hearing loss. For example, they may experience frustration and a reduced self-esteem due to the difficulties associated with understanding speech [18,19]. Because of the negative stereotypes and communication difficulties associated with ARHL, some older adults avoid social gathering, including family reunions. This type of social isolation may result in loneliness, depression and other forms of physical and mental health conditions [20]. Also, fatigue caused by hearing difficulties can have secondary consequences such as increased stress and tension among couples and other family members [21]. There is an increasing amount of literature indicating an association between hearing loss and cognitive decline [22–24]. Specifically, the prevalence of hearing loss is higher among people with dementia than among older adults who do not have cognitive problems. There is ample evidence to confirm that the presence of hearing loss may have negative effects on the quality of life and health of spouses and other family members who may not have hearing problems, [25–28]. In short, the presence of hearing impairment can have a negative impact on several health conditions as well as one’s quality of life.

## The Speech Communication Chain and Sources of Communication Breakdown

**FIGURE 2:**
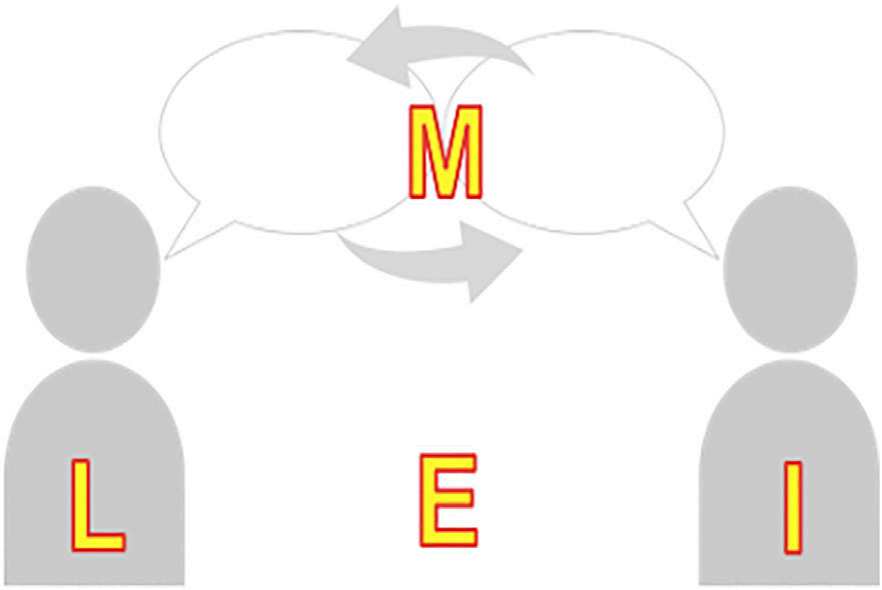
The LIME speech communication model. Legend: This figure was adapted from Lacerda et al. [31].

The LIME model is a simplified illustration of the speech communication chain [29]. In line with the International Classification of Functioning, Disability and Health [30], this model can be used to identify potential sources of communication breakdowns during conversations between two or more individuals (see Figure 2). The LIME model outlines factors associated with the four main elements involved in speech communication: 1) the Listener, 2) the Interlocutor, 3) the Message and 4) the Environment. All four elements are equally important in the communication process. Likewise, factors associated with any one of these four elements may create an obstacle to communication and cause a communication breakdown. A communication breakdown is defined as a failure to exchange information, resulting in a lack of communication [29]. Whereas the four elements of the simplified speech communication chain can constitute obstacles to communication, manipulating one or more of the elements can serve to overcome or avoid a communication breakdown and facilitate a verbal exchange between two or more individuals.

A communication breakdown occurs when the listener (L), a person with hearing loss, does not understand the intended spoken information transmitted by the interlocutor (I). To understand speech, the listener must detect and recognize the relevant speech elements, use working memory to store the information and apply other cognitive functions to decode the information and generate an appropriate response [32]. The listener’s psychological state (e.g., anxiety and stress level) may contribute to a communication breakdown. It is reported that older adults’ performance on memory tasks are sensitive to their level of stress [33]. The cognitive resources required to address the increased level of anxiety and stress may cause speech understanding difficulties. A communication breakdown may also occur because the interlocutor’s (I) speech is too soft, accentuated or distorted.

The message itself (M) can constitute the source of a communication breakdown. For example, a communication breakdown would occur if the listener is not knowledgeable of the vocabulary (e.g., medical terminology), language structure or cognitive concept being expressed by the interlocutor. Simplifying the language is a helpful strategy to use when communicating with an older person who has difficulty understanding the intended message.

Finally, and very importantly with regards to communication, the environment (E) in which a conversation takes place may constitute a facilitator or an obstacle to a fluent verbal exchange. For example, an appropriate source of lighting directed toward the interlocutor’s face will make it easier for the listener to extract the visual speech cues from the communication partner (i.e., the use of speechreading, facial expressions). On the other hand, a source of light emanating from behind the interlocutor may create a lighting contrast that makes it difficult to see the details of the talker’s face.

Related to speech communication, the presence of background acoustic noise typically constitutes the single most important obstacle to fluent conversations. Noise may be defined as any unwanted signal. For example, when two or more persons are having a conversation in a cafeteria, the sound generated by the other persons conversing and the clanging of utensils and dishes are considered to be sources of noise. The primary effect of background noise is a decrement in the speech signal-to-noise ratio (SNR), that is the difference in the level of the intended acoustic signal relative to the level of the background noise. For young normal hearing adults, a SNR of at least +1 dB is required to ensure good speech understanding [34]. Older adults with hearing loss require a SNR of approximately +15 dB to optimize speech understanding [35]. Similarly, reverberation may have a deleterious effect on speech understanding [36]. Reverberation can be defined as the persistence of a sound after its source has stopped, caused by the reflection of the sound in a closed space. In a typical closed environment, the speech signal arriving at the listener’s ear will be comprised of the speech signal emanating directly from the talker as well as numerous renditions of the same speech signal arriving at the ears after being reflected from the ceiling, floor and walls. Hence, there is a slight delay (in the order of milliseconds) between the direct signal and the reflected signals. This causes a “smearing” of the speech signal perceived by the listener. It should be noted that few communication settings offer a SNR and reverberation time that are optimal for communication.

All communication breakdowns can be accounted for by an obstacle that occurs at one or more of the fours elements of the LIME model of speech communication. Similarly, strategies that are effective in avoiding or repairing a communication breakdown can be associated with one of the four elements of the LIME model.

## Pandemic Specific Issues

With the COVID-19 outbreak in countries around the world, the WHO and public health agencies recommend the use of many measures to reduce the transmission of the virus [1]. Some of these recommendations may make communication more arduous for older individuals with hearing loss, whether or not they use hearing aids.

### Physical Distancing

Physical distancing (e.g., keeping a distance of 2 m between the communication partners) is not optimal for communication, especially in the presence of background noise and when one of the communication partners has hearing loss or visual impairment. The level of a sound decreases as a function of the distance from the source of the sound. In a free field (i.e., when there is no reverberation), the level of an acoustic signal decreases by 6 dB each time the distance from the sound source is doubled [37]. Under normal conditions, the physical distance between two individuals conversing is about 1 m. When the 2 m social distancing rule is applied, the speech signal from the conversational partner may be sufficiently attenuated to create speech understanding difficulties for the person receiving the message.

### Masks, Face Shields and Transparent Partitions

The results of recent investigations have revealed that the surgical mask acts as an acoustical low-pass filter, reducing the level of high-frequency sounds (between 2 and 7 kHz) [38,39]. The overall sound attenuation recorded when using a surgical mask is approximately 4 dB; an attenuation of 6–12 dB was observed when a N95 mask is used. Although face shields and masks with windows allow for speechreading, they attenuate the speech signal more than opaque cloth masks, with an attenuation of 11–14 dB [38]. Studies that have investigated the acoustic properties of facial protective devices confirm that the use of masks and shields can have a considerable negative impact on speech understanding as well as deleterious effects on secondary and tertiary issues related to hearing and communication (e.g., anxiety, fatigue and stress). In Italy, a research team investigated the impact of protective measures against COVID-19 among individuals with hearing loss who were hospitalized [40]. More than 85% of the patients questioned reported communication difficulties related to the implementation of protective measures. Related to wearing a mask, eliminating the possibility of lipreading was identified as the main source of communication disturbance in 55% of the participants questioned.

Also, the use of face masks may have detrimental psychological effects, which could impact communication as well as the quality of health services provided. For instance, Metha et al. (2020) reported that face masks decrease the feeling of trust between the patient and the health care provider [41]. Further, it is more difficult for the communication partners (e.g., the patient and the professional) to decode emotions when the only facial expression available is the other person’s eyes and eyebrows.

Three of the strategies most recommended for countering the deleterious effects of background noise are: 1) eliminate or reduce the background noise (i.e., improve the SNR), 2) provide visual cues so that the listener can use speechreading to complement the less than ideal acoustic speech signal and, 3) reduce the distance between the two interlocutors. However, pandemic protective measures, such as the use of a face mask and physical distancing, preclude the use of some of those strategies.

### Communication Technologies

With the COVID-19 pandemic, there has been a sudden increase in the use of telehealth. Many health care professionals now rely on the telephone or videoconferencing to provide health care services to their patients. Although these communication technologies serve to reduce the risk of transmitting an infectious disease, they can be obstacles to effective communication for older adults, especially for those with hearing difficulties. The primary obstacles to distance communication include (but may not be limited to): the quality of the communication device (i.e., telephone, computer, tablet, smartphone) and Internet connection, the quality of the acoustic signal (mainly influenced by the quality of the loudspeakers or earphones used), a poor temporal synchrony between the acoustic and the visual signals when videoconferencing and the lack of access to visual cues while using the telephone. These factors may interfere with the fluency of speech communication and lead to communication breakdowns. It is also important to keep in mind that although older adults report mainly positive attitudes toward the use of technologies [42], they may have less comfort and competence with the use of high-technology communication devices such as computers, smartphones and tablets [43].

## Strategies to Optimize Communication

As mentioned previously, communication is a shared responsibility between the listener and the interlocutor. Given this premise, using appropriate communication strategies is the responsibility of all communication partners. Communication strategies may be defined as the set of attitudes, knowledge, actions and requests that can be used to promote more effective communication [29]. A communication breakdown occurs when the listener does not comprehend the message provided by the interlocutor. Applying appropriate communication strategies makes communication more efficient, allows for a more pleasant communication experience and is beneficial for all the people involved in the conversations. There are three main categories of communication strategies [4,29]: 1) anticipation strategies, 2) repair strategies and 3) maintenance strategies.

### Anticipation Strategies

Anticipation strategies are employed to avoid or minimize communication breakdowns. They aim to anticipate difficult situations. For example, closing the door leading to a noisy hallway before conversing with an older adult with hearing loss would be an appropriate anticipation strategy. Likewise, verifying that a patient’s hearing aids are functioning properly before conducting a medical case history would be classified as an anticipatory strategy. Typical anticipation strategies include informing others about difficulty hearing, modifying the physical environment and informing about the subject and the terminology before the conversation. Vignette 1 illustrates examples of anticipation strategies.

VIGNETTE 1 **Anticipation Strategies**

Mrs. A was recently hired by a nursing home. She previously worked as a nurse in a private clinic, but since the COVID-19 outbreak, she decided to re-orient her career. Today, she must attend to Mr. B, a male patient of 95 years-old, who requires blood work. Before entering Mr. B’s room, Mrs. A puts on her complete protective gear, including a surgical face mask and a plastic face shield. Mr. B is watching television, with is back to the door. The lights are lightly dimmed, and Mr. B is not wearing is hearing aids, which are on the night table. While she prepares the equipment needed, Mrs. A explains to Mr. B that she will draw blood. Mrs. A then takes a seat beside Mr. B in order to begin the blood test. It is only then that Mr. B, notices her presence in the room. He is surprised and startled. He does not understand what this woman, wearing a complete protective attire, is doing in his room. Because of the face mask and shield, Mrs. A's speech is muffled and incomprehensible.

To optimize communication in this setting, Mrs. A could have used anticipation strategies:

1. Flashed the lights to warn Mr. B that someone was entering the room or walked in his line of sight and greet him good morning.
2. Turned-on the lights to ensure good lighting, closed the bedroom door and turned off the television to reduce ambient noise as much as possible.
3. Asked Mr. B to verify if the hearing aid batteries were operational and then put on his hearing aids (assisting him if help was needed).
4. Asked if Mr. B understood correctly or if he needed clarifications.

### Repair Strategies

Repair strategies are actions taken to restore the conversational flow after a communication breakdown occurs. For example, repeating the message while using louder and clearer speech is a form of repair strategy used to overcome a communication breakdown. Clear speech is characterized by a spoken signal that is slightly louder, articulated precisely, uttered at a slower than normal rate, and it includes the insertion of natural pauses between words, phrases and sentences. Although all of those modifications generate a signal that optimizes speech intelligibility, it is important not to exaggerate any of the suggested speech manipulations. The most appropriate repair strategies to use will depend on the source or the cause of the communication breakdown. Applying the LIME model (Listener, Interlocutor, Message or Environment) outlined earlier may be a good way to identify possible sources of a communication breakdown. For example, if it is determined that the breakdown is attributable to an inattentive listener, an efficient repair strategy would be to capture the listener’s attention before repeating the intended message. Typical repair strategies include asking to specify, clear speech, reformulating and using a synonym, elaborating, using non-verbal and body language, giving the context of the conversation and spelling or writing. Vignette 2 provides examples of repair strategies.

VIGNETTE 2 **Repair Strategies.**

Mr. M, a 70-year-old male with a severe hearing loss, comes in the pharmacy to pick up his prescribed medicine. The pharmacist (Ms. F) is wearing a face mask and is standing behind a plexiglass partition. The pharmacist reads Mr. M’s prescription and asks if he is familiar with the medication and if he has taken this medication before. Because of the background noise, the mask and the partition, the pharmacist’s voice is distorted and too soft for Mr. M to understand what she asked. He replies, somewhat inappropriately, that it was his family physician that prescribed this medication for him. Given his response, the pharmacist repeats (verbatim) what she asked him initially. After several unsuccessful repetitions (and slightly annoyed), the pharmacist finally gives up, and heads for the laboratory counter to prepare the medication. Upon her return, she explains what the medication is for and instructs Mr. M on the daily dosage he should take. Mr. M has difficulty understanding the technical terms used by the pharmacist. Furthermore, he misunderstands the instructions regarding the daily dosage. He understands that he should take two pills in the morning and at night. In fact, the pharmacist had instructed him to “take one pill in the morning, but not in the evening.” At the cash register, Mr. M does not hear when the pharmacist informs him of the cost of the medication. He simply gives her his credit card rather than ask her to repeat.

To optimize communication in this setting, the pharmacist could have used repair strategies:

1. Used clear speech (i.e., speak at a slower rate and slightly louder as well as articulate each syllable clearly without exaggerating) when providing information to Mr. M.
2. Stood facing Mr. M and used body language (eyes, eyebrows, gestures and body posture) to communicate more effectively with the face mask.
3. Used a pen and paper to write down the questions and instructions. Also, she could have pointed the window of the cash register to indicate the cost of the medication.
4. Avoided jargon or technical language when giving information to Mr. M about his medication, and used short and simple sentences.
5. Reformulated the instructions to facilitate Mr. M’s understanding, rather than simply repeating the same thing.
6. Ensure that Mr. M understood the instructions by asking him what dosage of medication he should take each day.

### Maintenance Strategies

Finally, maintenance strategies are used to demonstrate a person’s interest and active participation in the ongoing conversation. Nodding to signal to the communication partner that the message was understood is a common and effective maintenance strategy to use during a conversation. Common maintenance strategies include physical proximity, visual contact, facial expressions and providing confirmation and feedback. Vignettes 3 provides examples of maintenance strategies.

VIGNETTE 3 **Anticipation, Repair and Maintenance Strategies.**

Mrs. Y is a general nurse-practitioner. Because of the pandemic, in order to reduce social contacts and limit the spread of the COVID-19, many of her appointments are conducted by telephone. She conducts her interviews from her hospital office, which is adjacent to a general waiting area shared by several practitioners. Today is a particularly busy day in the clinic and the level of noise in the waiting room is quite high. Mrs. Y's 10-o’clock appointment is with Mr. D, a 65-year-old male with a known age-related hearing loss. Wanting to show her open attitude toward her patients and co-workers, Mrs. Y typically leaves her office door open even during appointments. Mrs. Y uses the hand-free option on her cellphone to call her patient. This allows her to take notes on the computer at the same time as she conducts the interview. Because she is running late in her appointments, Mrs. Y goes straight to the point and questions Mr. D on his symptoms. Mr. D struggles to understand Mrs. Y’s questions, because there seems to be a lot of echo and background noise coming from the telephone. Mrs. Y talks too quickly for Mr. D. to understand her. Consequently, for almost every question he is asked, Mr. D. needs several repetitions before he understands and answers the question.

To optimize communication in this setting, Mrs. Y could have used multiple strategies:

1. Shut the door of her office to reduce background noise and reverberation, that is being transmitted over the telephone line (anticipation strategy).
2. Used earphones with an integrated microphone with her telephone, or at least, not use the hand-free function in order to reduce the reverberation and the background noise (anticipation strategy).
3. Introduced herself before starting the interview, verified that Mr. D. could hear her well and then ask what the general reason for the consultation was (anticipation strategy).
4. Used alternate ways to seek the information sought rather than always repeating exactly the same thing every time Mr. D. asked for a repetition (repair strategy).
5. Used clear speech (repair strategy).
6. Considered using videoconferencing (thus allowing the use of visual cues) (maintenance strategy).
7. Verified with Mr. D that he was hearing well the questions and asked him to provide confirmation during the interview that he understood well (maintenance strategy).

### Summary of Communication Strategies

Based on the LIME model, Table 1 provides a summary of communication strategies that health care professionals can use in order to improve communication with patients that experience hearing difficulties either during a face-to-face conversation or while using the telephone or videoconferencing.

**TABLE 1:** Strategies for face to face, telephone and videoconferencing conversations based on the LIME communication chain.

	Listener	Interlocutor	Message	Environment
Shared (face to face and distance)	• At the beginning of the conversation, say that you are hearing impaired	• Use the eyes, eyebrows, hand gestures and body posture to communicate more effectively and to facilitate the understanding of the message	• If the other person does not understand, say in other words what you want to explain to him rather than repeating the same thing	• Make sure you have good lighting; make sure it is facing your face rather than behind you
	• Explain how the other person can help you: Speak slightly louder with good enunciation	• Make sure the other person understands what you told them by asking them if they understood correctly or if they need clarification	• Let the other person know the topic of the discussion in advance	• Reduce ambient noise as much as possible. When possible move to quieter spot or turn down the noise
	• Share what you heard (or think you heard)	• Allow time for the other person to express themselves	• Speak one at the time. Respect the speaking turn	
	• Include a friend or family member to ensure understanding or offer to come along to listen and take notes when a friend or family member has an appointment or meeting	• Speak a little louder and clearly while remaining natural; do not shout, as this can make it more difficult for the other person to read lips and distort speech	• Use short sentences and pause between each	
	• Make sure that you are wearing your hearing aids and that they are working	• Speak slower. Be understanding and stay calm	• Avoid jargon or technical language; use everyday speech	
	• Request rephrasing after two repeats			
		
Face to face	• Make sure that you are wearing your hearing aids and glasses	• Stand facing to the other person, even if you are wearing a mask	• When necessary, use pen and paper to write down what you want to say to the other person. Or, use the smartphone talk-to-text application to communicate	• Use a transparent face mask to allow lip-reading
	• If necessary, use a personal listening device while following appropriate infection control protocols	• Get the other person’s attention by saying their name or gesturing first	• Make the instructions accessible in writing (e.g., a sign stating to show the medical card at the reception)	• Be mindful of distance. As distance increases, sound levels decrease, and visual cues are more difficult to see
		
Distance (telephone and videoconferencing)	• Use assistive listening devices (e.g., connect hearing aids with the computer or the telephone)	• Use mute mode if you are not speaking to reduce background noise	• When possible, favor the use of written communication (e.g., email) for confirmation of appointments	• Speak in a room that is less reverberant
	• Make sure to have a good internet or phone reception	• Make sure to have a good internet or phone reception	• Ask that each speaker announce their name before they begin speaking	
	• Use proper earphones and microphone	• Before the start of the consultation, confirm with the other person that he can hear and understand you	• Use speech-to-text captioning when available	
		• Do not use the hands-free function when you are talking to the other person	• Consider using a speech to text app (e.g., my call to text)	
		• Do not cover your mouth with your hands	• Consider whether a relay service would be beneficial	

## Conclusion

In the context of a pandemic, effective communication may be more at risk than usual because protective and preventive measures are applied while providing health care services. Protective measures that may negatively impact speech communication include physical distancing, the use of face masks and shields as well as the increased use of telephone and videoconferencing for distance communication. Older adults, especially those with hearing loss, are particularly at risk of experiencing communication breakdowns and increased social isolation. Using hearing aids may help overcome the loss of signal intensity. However, they do not restore normal hearing and do not improve the loss of clarity. Communication strategies (anticipation, repair and maintenance strategies) should be used by all partners involved in the communication situation, including the health care professional, in order to maintain good interactions and prevent communication breakdowns. In almost every country, there are professionals trained to provide hearing health care services (e.g., audiologists, otologists/otorhinolaryngologists, hearing aids specialists and speech-language pathologists). Some of these professionals are responsible for the prevention, assessment and treatment of hearing disorders and provide hearing rehabilitation services for individuals with hearing difficulties. For more information concerning any issues related to hearing and communication, it is suggested that the health care professional consult the accredited hearing health care professional in their jurisdiction.
